# Pathogenic/likely pathogenic mutations identified in Vietnamese children diagnosed with autism spectrum disorder using high-resolution SNP genotyping platform

**DOI:** 10.1038/s41598-024-52777-y

**Published:** 2024-01-29

**Authors:** Duyen T. Bui, Anh N. V. Ton, Chi T. D. Nguyen, Son H. Nguyen, Hao K. Tran, Xuan T. Nguyen, Hang T. Nguyen, Giang L. T. Pham, Dong S. Tran, Jillian Harrington, Hiep N. Pham, Tuyen N. V. Pham, Tuan A. Cao

**Affiliations:** 1 Genetica Research Foundation, National Innovation Center, Hanoi, Vietnam; 2 Pediatric Center Hue Central Hospital, Hue City, Thua Thien Hue Vietnam; 3 Hue University of Medicine and Pharmacy, Thua Thien Hue, Vietnam; 4 Gene Friend Way Inc, San Francisco, USA

**Keywords:** Biotechnology, Genetics, Biomarkers, Neurology

## Abstract

Among the most prevalent neurodevelopmental disorders, Autism Spectrum Disorder (ASD) is highly diverse showing a broad phenotypic spectrum. ASD also couples with a broad range of mutations, both de novo and inherited. In this study, we used a proprietary SNP genotyping chip to analyze the genomic DNA of 250 Vietnamese children diagnosed with ASD. Our Single Nucleotide Polymorphism (SNP) genotyping chip directly targets more than 800 thousand SNPs in the genome. Our primary focus was to identify pathogenic/likely pathogenic mutations that are potentially linked to more severe symptoms of autism. We identified and validated 23 pathogenic/likely pathogenic mutations in this initial study. The data shows that these mutations were detected in several cases spanning multiple biological pathways. Among the confirmed SNPs, mutations were identified in genes previously known to be strongly associated with ASD such as *SLCO1B1, ACADSB, TCF4, HCP5, MOCOS, SRD5A2, MCCC2, DCC*, and *PRKN* while several other mutations are known to associate with autistic traits or other neurodevelopmental disorders. Some mutations were found in multiple patients and some patients carried multiple pathogenic/likely pathogenic mutations. These findings contribute to the identification of potential targets for therapeutic solutions in what is considered a genetically heterogeneous neurodevelopmental disorder.

## Introduction

### Autism spectrum disorder

Autism spectrum disorder (ASD) is known to be a heritable and heterogeneous group of neurodevelopmental phenotypes that manifests itself via numerous social, emotional, communicative, and behavioral challenges^1^. The origin of ASD is not fully understood, and evidence suggests that both genetic and environmental factors are involved^2,3^. Although most everyone in the general population carries almost all the genetic risk factors for autism, the disorder, as per its modern terminology, lies on a spectrum or continuum, with only the extreme tail of individuals expressing its defined signs to a more notable degree^4^. Antaki et al. have postulated that the phenotypic spectrum of ASD is correlated to multiple genetic factors, parental factors, and gene-by-sex effects. The authors also underscored that their research showcases the importance that rare and common variations, in combination, yield greater contributions to the development of ASD than alone^5^. Individuals with ASD can display various aspects of cognitive difficulties, with the prevalence of intellectual disability in conjunction with ASD estimated to be 3.8 in 1000 in an Australian sample for births from 1993 to 2005^6^.

### Prevalence among east asians

Globally, the World Health Organization^7^ estimates that 1 in 100 children have ASD with a consistently higher prevalence in males^8^. In 2018, researchers in the U.S. estimated that 1 in 44 children aged 8 years had ASD. Among Asian Americans, the prevalence of ASD appears to be on the rise due to increased visibility and public awareness of the disorder^9^.

Often, the prevalence of ASD has been reported to be lower in East Asia than in Europe or North America. However, this discrepancy may be due to methodological differences between studies. In fact, modernized research in China indicates that the prevalence rates are comparable between the geographic locations^10^. More broadly, it’s estimated that the prevalence of ASD in East Asia stands at 0.51% and is likely increasing^11^.

### Diagnostic and therapeutic challenges for East/Southeast Asians

Numerous aspects of East Asian culture are thought to influence clinical diagnosis and treatment for children with ASD; as well as the way the clinician might interact with an East Asian parent in the context of ASD^12^. Clinicians must consider the culture and the stigma that may be associated with autism and how parents of autistic children might view the disorder.

Many of these same, and other, cultural factors can also affect parental care and well-being. For instance, stress among parents with children diagnosed with ASD may partly stem from unique cultural thoughts (parental stress due to cultural factors, parents’ psychopathological symptoms, problem behaviors in ASD children, caregiver burden and more were identified) present in East Asia and Southeast Asia that are not found in other cultures to the same degree^13^. Furthermore, we see an increase in diagnostic rate in more developed countries in the regions such as Singapore, Philippines (1–1.1%) compared to Vietnam, Thailand (~ 0.7%) and Laos, Cambodia, Brunei (~ 0.5%). This rate does not correlate geographically, indicating that among East Asian and Southeast Asian, there can be a lack of proper understanding of the diagnosis and treatment of children with ASD starting from parental recognition to start doctor’s visit^14^.

This points to two things. First, there is a need to utilize, in concert with existing methodologies, standardized genetic screening techniques for the diagnosis of ASD. Techniques that may have been previously underutilized due to cultural and socioeconomic differences, even among Asian populations. Second, to increase the rate of appropriate diagnosis and treatment, there is a need to properly educate clinicians and parents on ASD in the context of specific Asian cultural factors.

## Methods

### Sample collection

Parents of autistic children were present during sample collection at Hue Central Hospital. Children in this study were diagnosed using the DSM-IV criteria (American Psychiatric Association (2000)-Diagnostic and statistical manual of mental disorders (4th edition)). Participant’s screening results are in supplemental Table 3. Saliva samples were stored at room temperature and subjected to automated DNA extraction and processing. A total of 254 saliva samples were collected using the Oragene•ONE ON-600 (Dnagenotek, Canada).

### Ethics declaration

Prior to the start of this study and enrollment of research participants, the Ethics committee of Hue Central Hospital in Vietnam approved this study. All the methods were performed in accordance with the relevant local guidelines and regulations.

Prior to collection of the saliva samples, written informed consents were signed by parents of 254 children diagnosed with Autism Spectrum Disorder allowing the authors of this study to use the saliva samples and all the related medical data for research, publications, and biobanking purposes.

### DNA extraction

The genomic DNA was extracted from the collected saliva using Chemagic Prime™ Robot. The process is entirely automated using chemagen patented M-PVA Magnetic Bead technology for DNA and RNA purification with liquid handling to provide high throughput automated isolation of ultra-pure nucleic acids. The process is monitored in accordance with the Quality Control of ISO/IEC 17025.

### Genotyping with The GFWv3 custom high-resolution arrays

The Axiom workflow was used on GeneTitan instruments (Manufacturer: Thermo Fisher Scientific, catalog number: 00-0373, Model: GeneTitan MC) with wrappers for Analysis Power Tools (APT)–Genotype–quality control tools (apt-geno-qc) and genotype calling tools (apt-probeset-genotype). A tool for SNP metric calculations and a tool to convert the output into Plink format for downstream genomics analyses were used. Samples were registered in a custom file format for a batch of 96 “.CEL” files from a single Axiom plate and a few auxiliary file formats specific to APT tools to facilitate the file selection process in Galaxy. The Galaxy workflow starts with receiving its input of “.CEL” and “.ARR” files from the instrumentation computer. It proceeds with extracting the “.CEL” files and executing the quality control tool with a user-specified Dish-Quality Control (QC) threshold (by default 0.82). The names of the samples that have passed the QC are passed on to the genotyping tool, along with the “.CEL” dataset, for the first round of genotyping. The output from this first round contains, among other metrics, the call rates for each sample. The samples with a call rate above a user-specified threshold (by default 97%), along with the “.CEL” dataset again, is input for the second iteration of genotyping. The final genotype calling report is then annotated with the phenotype data and converted into Plink format, and simultaneously processed by the SNP metrics tool to calculate such statistics as Call Rate (CR), Fisher’s linear discriminant (FLD), FLD calculated for the homozygous genotype clusters (HomFLD), Minor Allele count.

The GFWv3 custom array is a High-resolution Affymetry SNP array consisting of 2.5 million (2.5 M) probes to assay for SNPs and CNVs with 800.000 direct targets and two million more with imputation. We designed and validated this array by both inter-assays (reproducibility >  = 99.8%) and intra-assays (reproducibility >  = 99.8%). Manufacturer: Thermo Fisher Scientific. There are 3 parts of each kit: Axiom™ GFWv3 96 well Plate: part number 551159; Axiom™ GeneTitan Consumables Kit: part number 901606 and the Axiom™ 2.0 Reagent Kit: part number 901758.

### Variant validation

Identified variants were validated by Sanger sequencing and Amplification-refractory mutation system polymerase chain reaction (ARMS-PCR). Each unique mutation found in this study was validated using Sanger sequencing. If there are several samples found to carry the same mutation, we did Sanger sequencing to verify the mutation identified in one sample and in parallel, ARMS PCRs for the other samples carrying the same mutation. This method became a more cost-effective and accurate workflow as we expanded the analyses to more common variants. The principle of validation of identified variants with ARMS-PCR is shown in Supplemental Fig. 1. The list of primers designed for PCR and Sanger sequencing is included in Supplemental Table 1.

These oligos were designed for identified variants by using the Primer-BLAST tool of NCBI (https://www.ncbi.nlm.nih.gov/tools/primer-blast/) and PRIMER1 on the website http://primer1.soton.ac.uk/primer1.html. The same DNA samples used for genotyping in this study were also used for the validation process. PCR amplification, Sanger sequencing, and ARMS PCR for variant verification.

## Results

### Demographic and clinical characteristics of the research participants in this study

All 254 children recruited as research participants were from Central Vietnam and are current patients of Hue Central Hospital. We have not collected and analyzed DNA from the parents as the initial results showed all mutations found were heterozygous. The children were diagnosed with ASD using the DSM-IV. The male/female ratio in this study is 5.86, (Table 1). This is higher than previously estimated ratio in the general population which is closer to 4^8,11,15–17^. This could be due to several factors such as those willing to participate in the study skewing the ratio to more males than expected. Furthermore, the fact that females often being diagnosed with ASD less or later than males or having different, less obvious symptoms compared to males^18^ might contribute to this result.

**Table 1:** Demographic and clinical characteristics of the participants in this study.

	Female	Male
Gender	37	217
Age in years (average (SD))	3.4 (1.7)	3.8 (2.3)
Normal (vaginal) birth	16	101
Cesarean-section or assisted delivery	21	116
Full-term birth	35	198
Premature birth	2	19
Delayed speech	37	217

All 254 children diagnosed with ASD and ASD-like behavior are shown in this table by gender, age (shown in average years old, and Standard Deviation (SD) in parenthesis). Delayed speech means impairment of verbal communication, one of the diagnostic criteria for ASD outlined in DSM-IV.

Among the 254 samples collected, four samples were degraded yielding minimal amount of DNA or low-quality DNA. The families decided not to recollect these four samples. For the 250 samples that passed QC, the genotyping results were analyzed using the Axiom analyses Suite 5.1 (Thermo Fisher).

All the children in this study have speech delays characterized by no word being spoken by the age of 16 months and other communication issues such as not responding to their name when being called, avoiding eye contact or avoiding interacting with others, as reported by their parents (Table 1 and Sup. Table 3).

Mutations identified were categorized as pathogenic/likely pathogenic according to Clinvar database (https://www.ncbi.nlm.nih.gov/clinvar/). A total of 23 pathogenic/ likely pathogenic mutations were identified in this study including 12 missense mutations, 5 stop-gained mutations, 3 frameshift mutations, 1 splicing mutation, and two in non-coding transcripts (Table 2). A few variants showed conflicting interpretations (pathogenic to benign). For instance, rs1799990 in the *PRNP* gene was classified as pathogenic/risk factor/ likely benign. However, based on the high frequency of occurrence of this SNP (also shown in this study), it was classified as a likely benign variant.

**Table 2:** Pathogenic/likely pathogenic variants identified in this study.

No	Gene	RSID	Genome changes	Amino acid change	Types of changes	Pathogenicity
1	*RIPK1*	rs116040763	NC_000006.12:g.3113257C>T	NP_001341859.1:p.Thr645Met	Missense variant	Pathogenic
2	*SLCO1B1*	rs200994482	NC_000012.12:g.21224840G>A	NA	Splice donor variant	Pathogenic
3	*ACADSB*	rs779015128	NC_000010.11:g.123043110delC	NP_001317103.1:p.Pro147fs	Frame shift	Pathogenic
4	*TCF4*	rs587784464	NC_000018.10:g.55350904G>A	NP_001356514.1:p.Arg132Ter	Stop gained	Pathogenic
5	*HCP5*	rs2395029	NC_000006.12:g.31464003 T>G	NR_040662.1	Non coding transcript variant	Pathogenic; risk factor
6	*KAT6A*	rs139494583	NC_000008.10:g.41792077C > T	NP_006757.2:p.Glu1221Lys	Missense variant	Pathogenic
7	*MOCOS*	rs750896617	NC_000018.10:g.36260092C > T	NP_060417.4:p.Arg776Cys	Missense Variant	Pathogenic
8	*SRD5A2*	rs9332964	NC_000002.12:g.31529325C>T	NP_000339.2:p.Arg227Gln	Missense variant	Pathogenic/likely pathogenic
9	*CUBN*	rs143944436	NC_000010.11:g.16940152G>A	NP_011518011.1:p.Arg1810Ter	Stop gained	Pathogenic/likely pathogenic
10	*MCCC2*	rs119103221	NC_000005.10:g.71635176C>G	NP_001350076.1:p.Pro272Arg	Missense variant	Pathogenic
11	*GJB2*	rs80338943	NC_000013.11:g.20189349del	NP_003995.2:p.Leu79fs	Frame shift	Pathogenic
12	*TACR3*	rs764659822	NC_000004.12:g.103658260G>A	NP_001050.1:p.Thr231Ile	Missense Variant	Likely pathogenic
13	*PRNP*	rs1799990	NC_000020.11:g.4699605A > G	NP_001073590.1:p.Met129Val	Missense variant	Pathogenic/risk factor/likely benign
14	*DCC*	rs775565634	NC_000018.10:g.53339808G>A	NP_005206.2:p.Val754Met	Missense variant	Pathogenic
15	*GJB2*	rs111033204	NC_000013.11:g.20189282_20189283del	NP_003995.2:p.His100fs	Frame shift	Pathogenic
16	*LOC107987057*	rs2814707	NC_000009.12:g.27536399C>T		Non coding transcript variant intron variant	Uncertain significance
17	*ZGRF1*	rs61745597	NC_000004.12:g.112623837G > T	NP_060862.3:p.Leu48Met	Missense variant	Benign
18	*FAM98C*	rs201037487	NC_000019.10:g.38407003C>T	NP_777565.3:p.Arg282Ter	Stop gained	Likely pathogenic
19	*ZGRF1*	rs76187047	NC_000004.11:g.113506711C>T	NP_060862.3:p.Glu1363Lys	Missense variant	Benign
20	*SCN9A*	rs12478318	NC_000002.12:g.166277030 T>G	NP_002968.1:p.Met932Leu	Missense Variant	Uncertain significance
21	*PRKN*	rs751037529	NC_000006.12:g.161785793C>G	NP_004553.2:p.Gly284Arg	Missense variant	Pathogenic
22	*SLC3A1*	rs200483989	NC_000002.12:g.44286074C>T	NP_000332.2:p.Arg270Ter	Stop gained	Pathogenic
23	*PYGM*	rs114073621	NC_000011.10:g.64751346G>A	NP_005600.1:p.Arg650Ter	Stop gained	Pathogenic

NC, NP, NR is the reference sequence based on a Chromosome, Protein (amino acid) sequence, Non-protein-coding RNA, respectively; g is genomic reference sequence; c is coding DNA reference sequence; For example: g.161785793C > G indicates substitution of the C nucleotide at genomic position g.161785793 with a G.

*NA* not available.

Multiple pathogenic mutations were identified in single individuals. Several cases with very severe ASD diagnosis were found to be the carriers for multiple mutations. For instance, AUT4875, is the carrier of 4 different mutations, two in the *ZGRF1* gene, one in the *SCN9A* gene and another in the *HCP5* gene; AUT4870 carries mutations in the *RIPK1* and *SCN9A* gene (Table 3).

**Table 3:** Genes in which pathogenic/likely pathogenic mutations were identified in this study.

AUT number	Gene	Related diseases
AUT4870	*RIPK1* (rs116040763)	Immunodeficiency. Immune dysregulation-inflammatory bowel disease-arthritis-recurrent infections syndrome, primary immunodeficiency. The fragile X mental retardation protein (FMRP) affects multiple steps of mRNA metabolism during brain development and in different neoplastic processes. FMRP binds receptor-interacting protein kinase 1 (RIPK1) mRNA, suggesting that FMRP acts as a regulator of necroptosis pathway through the surveillance of RIPK1 mRNA metabolism.
AUT4650	*SLCO1B1* (rs200994482)	Rotor syndrome. Associated with the efficacy and pharmacokinetics of many drugs. Mutations in this gene were found in GWAS study for autism-affected families
AUT4864 AUT4712	*ACADSB* (rs779015128)	Deficiency of 2-methylbutyryl-CoA dehydrogenase 2-methylbutyryl-CoA dehydrogenase deficiency associated with autism and mental retardation. Affected patients were diagnosed because they presented clinically with seizures and psychomotor delay and had increased urinary excretion of 2-methylbutyryl glycine. The brain, where isoleucine is oxidized as fuel, is primarily affected in these patients.
AUT1034	*TCF4* (rs587784464)	Pitt-Hopkins syndrome, intellectual disability (ID) and autism spectrum disorder (ASD)
AUT1038 AUT4693 AUT4656 AUT4845 AUT4666 AUT4720 AUT5013 AUT4840 AUT4896 AUT4902 AUT4905 AUT4875 AUT4855 AUT4695	*HCP5* (rs2395029)	Highly associated with systemic lupus erythematosus (SLE) and has a high risk of developing autism *HCP5* rs2395029 is also tightly linked with HLA-B*5701 (100%)^19^, a marker associated with autism and intellectual disability^20^.
AUT5003	*KAT6A* (rs139494583)	*KAT6A* is strongly linked to autism, microcephaly and global developmental delay. In most instances, variations in the *KAT6A* gene occur spontaneously when there is no family history of the disorder (de novo variations).
AUT4862 AUT4641	*MOCOS* (rs750896617)	Downregulation of *MOCOS* is present in most (80%) ASD patients. Variants in *MOCOS* were shown to be associated with ASD.
AUT4653 AUT4632 AUT4712 AUT4997 AUT4652 AUT4889 AUT1029 AUT4935	*SRD5A2* (rs9332964)	Variants in *SRD5A2* known to affect enzymatic activity were found to associate with autistic-like traits in female subjects and also affect testosterone levels in boys with Asperger Syndrome
AUT4680 AUT4997	*CUBN* (rs143944436)	*CUBN* is a vitamin B12 related gene that is also associated with a blood disorder known as megaloblastic anemia and may develop neurological problems
AUT4679	MCCC2 (rs119103221)	3-methylcrotonyl-CoA carboxylase (MCC) deficiency (OMIM 210,200 and 210,210) is a defect in the catabolism of the amino acid leucine. Mutations in *MCCC2* had been associated with developmental outcomes including mental retardation and ASD
AUT4897 AUT4930 AUT4672 AUT4741	*GJB2* (rs80338943)	More prevalent variants are found in Asians compared to Caucasians that are related to non-syndromic hearing loss. *GJB2* encodes the connexin 26 protein. In the cochlea, Kikuchi et al. 1995 suggested that CX26-containing gap junctions maintain K+homeostasis by keeping K+away from the hair cells during auditory transduction^21^. Many of the mutations in *GJB2* gene have been reported to be the cause mutation for hearing impairment^22^
AUT1036 AUT4846	*TACR3* (rs764659822)	This gene belongs to a family of genes that function as receptors for tachykinins. *TARC3* mutation may cause Hypogonadotropic hypogonadism, it is associated with non-reproductive phenotypes, such as anosmia, cleft palate, and sensorineural hearing loss Neurokinin B (endogenous ligand of TACR3) is involved in the pathogenesis of Parkinson’s disease by interacting with brain dopaminergic transmission
AUT4653 AUT4626 AUT4912 AUT4895 AUT1036 AUT4845 AUT4847 AUT4703 AUT4862 AUT1045 AUT4851 AUT4651 AUT1054 AUT4702 AUT4935 AUT1050 AUT4850 AUT4991 AUT4855 AUT4624 AUT4657 AUT4648	*PRNP* (rs1799990)	Creutzfeldt-Jakob Diseases; Prion Gene that links to Prion diseases; a diverse group of neurodegenerative conditions
AUT4861 AUT4913 AUT4988	*DCC* (rs775565634)	*DCC* genes play an important role in synaptic function and plasticity in the central nervous system^23^, especially in the Corpus callosum. Haplotypes in *DCC* are associated with ASD susceptibility.
AUT4861	*GJB2* (rs111033204)	Encodes the connexin 26 protein. In the cochlea, Kikuchi et al., 1995 suggested that CX26-containing gap junctions maintain K+homeostasis by keeping K+away from the hair cells during auditory transduction^21^. Many of the mutations in *GJB2* gene have been reported to be the cause mutation for hearing impairment^22^.
AUT1054 AUT4917 AUT4861 AUT4935 AUT4839 AUT4635 AUT1062 AUT4688 AUT4998 AUT4727 AUT4706 AUT4737 AUT4632 AUT4912 AUT4886 AUT1020 AUT4987 AUT4714 AUT4666 AUT4894 AUT0932 AUT4920 AUT1052 AUT4934	*LOC107987057* (rs2814707)	rs2814707 associated with ALS risk. ALS is a neurodegenerative disorder that can lead to fatal paralysis.
AUT4875 AUT4861 AUT1045 AUT4904 AUT4916 AUT4647 AUT1020	*ZGRF1* (rs61745597)	Childhood apraxia of speech; Speech-language disorder; *ZGRF1* is also related to Hot water epilepsy (HWE).
AUT4661 AUT4643	*FAM98C* (rs201037487)	*FAM98C* is an ASD candidate gene^24^
AUT4875 AUT4861 AUT1045 AUT4904 AUT4916 AUT4647 AUT1020	*ZGRF1* (rs76187047)	*ZGRF1* is related to Hot water epilepsy (HWE)
AUT4627 AUT4665 AUT4646 AUT5009 AUT4876 AUT1054 AUT4917 AUT4994 AUT4870 AUT4715 AUT4902 AUT4835 AUT4698 AUT4875 AUT4862 AUT4823 AUT4630 AUT4878 AUT4859 AUT4856 AUT4634	*SCN9A* (rs12478318)	*SCN9A* is highly expressed in the hypothalamus, a brain region that has been implicated in autism. Addition to the role in GABAergic neurotransmission, the role of *SCN9A* in autism might be mediated through changes in hypothalamic functions, which in turn can affect multiple hormonally regulated processes that are frequently disrupted in autism such as oxytocin-mediated social interactions.
AUT1041	*PRKN* (rs751037529)	*PRKN* is implicated in brain development. The loss of the gene in mice results in autistic-like behaviors, accompanied with altered neuronal activity, abnormalities in synapse formation and synaptic molecular composition.
AUT1039	*SLC3A1* (rs200483989)	Thirteen of these genes belong to the *SLC* family, of which *SLC1A3, SLC32A1* and *SLC38A7* are strong candidates for schizophrenia (SCZ). Most disease-associated genes in the gene sets belong to the *SLC* family, implying the important role of *SLC* in ASD and SCZ.
AUT1050	*PYGM* (rs114073621)	Glycogen storage disease V. Protein levels of *PYGM* and *RAC1*, a kinase that regulates *PYGM* activity, are reduced in the astrocytes in schizophrenia^25^.

*AUT* autism cases number.

In the two predominant databases for genes associated with ASD, SFARI and AutDB, only 9 mutations were previously reported among the total of 23 unique pathogenic/likely pathogenic mutations identified. Though, many of the identified genes identified are implicated in ASD and other neurological disorders as discussed below. All 23 of the pathogenic/likely pathogenic SNPs assessed were confirmed via orthogonal methods (i.e. Sanger Sequencing or ARMS PCR). There are variations in genes previously strongly associated with ASD such as *SLCO1B1*^26^, *ACADSB*^27^, *TCF4*^28^, *HCP5*^29^, *MOCOS*^30^, *SRD5A2*^31^ , *MCCC2*^32^, *DCC*^33^ and *PRKN*^34^. The ratio of males/ females among the samples with identified mutations is 3.93 (Sup. Table 3).

## Discussion

To date, hundreds of potential genetic alterations have been identified as associated with autism though with no defining cohort. What has been determined is that there is likely shared pathophysiology for neurodevelopmental disorders and that autism is along a continuum between intellectual disability and schizophrenia^35^. Since ASD is multigenic and heterogeneous and can occur in conjunction with other neurological conditions, it is difficult to discern the genes that are responsible for the disease phenotypes^36^. Previous studies showed consistent results of two classes of proteins, those involved in synapse formation and those involved with transcriptional regulation and chromatin-remodeling pathways^37^.

From 250 Vietnamese children diagnosed with varying degrees of autism spectrum disorder, our high-resolution SNP array data has identified both rare and common SNPs previously known to associate with ASD, we then validated these data and provided information regarding frequency among Southeast Asians.

Of the confirmed SNPs, there were 7 SNPs that were shared among several of the children tested, these were SNPs in *HCP5, SRD5A2, PRNP, ZGRF1, SCN9A* and *LOC107987057*. rs2395029 in *HCP5* has not previously been identified as an autism-associated variant though there is increasing evidence that immune-related genes, such as *HCP5*, and immune dysregulation are associated with neurodevelopmental disorders^29^. Recent studies have shown that *HCP5* rs2395029 is in complete linkage disequilibrium with HLA-B*5701^19^, which is a risk allele of intellectual disability^20^. Several alleles of *HLA* genes have been reported to be associated with autism, intellectual disability, schizophrenia^20^. Using Fisher Exact test, frequency of this mutation among the cases in this study is significantly higher than its frequency in the East Asian population (p-value = 0.000046) (Sup. Table 4). In terms of *SRD5A2*, a recent study found that ASD boys with rs9282858 mutation in this testosterone metabolism-related gene showed higher levels of restricted and repetitive behaviors^31^. In addition, multiple recent studies published on Clinvar have consistently concluded *SRD5A2* rs9332964 as pathogenic/likely pathogenic. Frequency of this mutation among the cases in this study is also significantly higher than its frequency in the general East Asian population (p-value = 0.00013) (Sup. Table 4). Recently studies showed that key proteins in the metabolism of ROS were downregulated in autistic people, including *PRNP*, marking *PRNP* as a potential biomarker of autism for early diagnostic purposes^38^.

*ZGRF1* encodes a protein with functions related to motor praxis and highly expressed in the cerebellum, raising the possibility that disrupted *ZGRF1* may interfere with cerebellar function^39,40^. Two *ZGRF1* variants detected as compound heterozygotes in 7 ASD patients in this study, rs61745597 and rs76187047, have been identified as the potential genetic causality of childhood apraxia of speech (CAS), which is prevalent in approximately 25–30% of children with ASD^41^. Both variants result in missense mutations, thereby it is logical to expect disruption of the *ZGRF1* protein in both instances. While CAS is a complex disorder, it is unlikely that *ZGRF1* would be the sole causative gene target, it is one more piece in the puzzle when narrowing down potential therapeutic targets for ASD and its associated disorders^40^. Mutations in the primary central nervous system sodium channels are associated with neurological, psychiatric, and neurodevelopmental disorders including autism. *SCN9A* has been indicated to be important for normal brain function and variants in this gene are involved in familial autism^42^. *LOC107987057* or *C9orf72* variants have been linked to several neurological disorders, including ASD^43^. However, Chi-square analyses showed that the frequencies of these 4 variants *LOC107987057* rs2814707, *ZGRF1* rs61745597, *ZGRF1* rs76187047 and *SCN9A* rs12478318 observed in our study are not significantly different from the East Asian population in the 1000 Genome Project 30x (p-value = 0.27, 0.82, 0.82, and 0.21 respectively) (Sup. Table 4). In addition, given the high frequency of the minor allele, the association of these variants to ASD should be reconsidered.

Confirmed variants in the following *GJB2* were seen in five or six children, respectively. *GJB2* is most known for its linkage in children with non-syndromic genetic sensorineural hearing loss and has not been identified previously as having an association with ASD^22^.

Confirmed variants in *RIPK1*^44^, *CAPN3*^45^, *KAT6A*^46^, *TACR3*^47^, *GJB2*^48^, *FAM98C*^24^, *PRKN, SLC3A1, CUBN,* and *PYGM* were seen in one or two children in this study. Interestingly, rs751037529 in *PRKN* has been identified as a pathogenic variant associated with early-onset Parkinson’s disease^49^. Also, *PRKN* knock-out mice show autistic-like behaviors, giving weight to *PRKN* as a potential candidate gene for ASD^34^. Previous studies have also shown that the disruption of genes that encode large amino acid transporters, like *SLC3A1*, increases the risk of ASD^50^. These abnormalities in large amino acid transporters can affect the utilization of certain amino acids and their availability during brain development, resulting in an increased risk of ASD. Along that line, rs143944436 in the *CUBN* gene identified in this study results in a premature stop codon resulting in a non-functional protein. The *CUBN* gene provides instructions for making a protein called cubilin which is involved in the uptake of vitamin B12 from food into the body, linking vitamins and their bioavailability as potential treatments for individuals with ASD.

Considering the vast number of inherited, common, and rare genetic variants that have been associated with ASD, the etiology is complicated, to say the least. This study specifically assessed a cohort of Southeast Asian children with varying degrees of ASD compared to a control population to identify those variants which may be potential diagnostic or therapeutic targets.

This study provides an initial step towards understanding the genetic underpinnings of ASD in Southeast Asian populations. We view these data as a contribution towards identifying the loci which contribute to ASD and we anticipate that some of these loci will eventually have sufficient evidence to become established robust ASD risk loci.

Some genetic variants correlate to a very high risk of disease while most do not. In its simplest terms, a polygenic risk score (PRS), sometimes called a polygenic score (PGS) or a genetic/genomic risk score (GRS), reflects the overall genetic predisposition to a disease based on the sum of all known and common variants linked to that disease^51^. This study has focused on pathogenic/likely pathogenic mutations. The next step, the study will be furthered with analysis of more samples, not only for SNPs but copy number variants and also the attribution of more common variants using the Polygenic Risk Score.

### Supplementary Information

Supplementary Information.

Supplementary Table 3.

Supplementary Table 4.

## Data Availability

Data generated and analyzed in this study are included in this article and its Supplementary Information. Our data are also deposited in ClinVar under accession numbers: SCV004013880—SCV004013903.
