# Supplementary material for: Pathogenic/likely pathogenic mutations identified in Vietnamese children diagnosed with autism spectrum disorder using high-resolution SNP genotyping platform
Source: Sci Rep. 2024 Jan 29;14:2360. doi: 10.1038/s41598-024-52777-y (PMC10825208; doi:10.1038/s41598-024-52777-y)
Supplement: Supplementary file 1 — Supplementary Information. [file 41598_2024_52777_MOESM1_ESM.docx]

Pathogenic/likely pathogenic mutations identified in Vietnamese children diagnosed with Autism Spectrum Disorder using high-resolution SNP genotyping platform

# Duyen T. Bui ^1,4^, Anh N.V. Ton^2,3^, Chi T.D. Nguyen^2^, Son H. Nguyen^2^ Hao K. Tran^2^, Xuan T. Nguyen^2^, Hang T. Nguyen^1,4^, Giang L.T. Pham^1,4^, Dong S. Tran^1,4^, Jillian Harrington^1,4^, Hiep N. Pham^2^ , Tuyen N.V. Pham^2^ and Tuan A. Cao^1,4^

# ^1^ Genetica Research Foundation, National Innovation Center, Hanoi, Vietnam

# ^2^ Pediatric Center Hue Central Hospital, Hue City, Thua Thien Hue, Vietnam

# ^3^ Hue University of Medicine and Pharmacy, Thua Thien Hue, Vietnam

# ^4^ Gene Friend Way Inc, San Francisco, USA

# Supplementary Information

Supplemental Table 1: Oligos designed for ARMS-variant-detection PCR, amplification PCR and Sanger Sequencing of identified variants

| **rsID** | **Gene** | **Primer name** | **Sequence (5'->3')** | **Length** | **Tm** | **Ta** | **%GC** | **Product Size (bp)** |
| --- | --- | --- | --- | --- | --- | --- | --- | --- |
| rs116040763 | *RIPK1* | F_RIP | AACTGGGCTTCACACAGTCT | 20 | 59.16 | 53 | 50 | 213 |
|  |  | R_RIP | TCCAGGGTTAGTTCTGGCTG | 20 | 59.02 |  | 55 |  |
| rs200994482 | *SLCO1B1* | F_SLC | TGTAGGGAGGGTACGAGTAGG | 21 | 59.51 | 55 | 57.14 | 745 |
|  |  | R_SLC | CAATCGAATACACACACATGC | 21 | 55.97 |  | 42.86 |  |
| rs779015128 | *ACADSB* | F_ACA | TCGTTTCATAATGCCCATGT | 20 | 55.41 | 50 | 40 | 693 |
|  |  | R_ACA | CTGGATCTTCTGCTGCTCAC | 20 | 58.35 |  | 55 |  |
| rs587784464 | *TCF4 ** | F_TCF | TCAGCTCCCAAATGCCCATT | 20 | 59.96 | 53 | 50 | 542 |
|  |  | R_TCF | GCAACCCAGGAACCCTTTC | 19 | 58.65 |  | 57.89 |  |
| rs2395029 | *HCP5 *** | F_HCP | GTTCCACACGAACTCCTCCT | 20 | 59.32 | 55 | 55 | 606 |
|  |  | R_HCP | TGCTCTCCGTGCAGTATCTT | 20 | 58.81 |  | 50 |  |
| rs139494583 | *KAT6A* | F_KAT | TCATCATCAGCGTCGTGGTC | 20 | 60.18 | 53 | 55 | 542 |
|  |  | R_KAT | GAGCCAGATACATCCACACCTT | 22 | 59.83 |  | 50 |  |
| rs750896617 | *MOCOS* | F_MOC | TGGGACTCTCACTGGAGATG | 20 | 58.14 | 52 | 55 | 725 |
|  |  | R_MOC | ACTGCCACAAAGTCCAACAA | 20 | 58.15 |  | 45 |  |
| rs9332964 | *SRD5A2* | F_SRD | TTCAATACAAGCCCAGCAAG | 20 | 56.31 | 51 | 45 | 342 |
|  |  | R_SRD | ACCTTCCGATTCTTCTGCAC | 20 | 57.9 |  | 50 |  |
| rs143944436 | *CUBN* | FM_CUB | AAATGCCACGGGTCACTTGGTGGTAT | 26 | 62.5 | 60 | 50 | 293 and 149 |
|  |  | F_CUB | TACTCTTCATAAATGTGGCCTGGAAGCCC | 29 | 61.7 |  | 48.3 |  |
|  |  | R_CUB | GGTTAACCTAGCATGTGATCCGGGAGAA | 28 | 61.4 |  | 50 |  |
| rs119103221 | *MCCC2* | FM_MCC | TTCCATACAATTCATCAGCAGGAAATATAC | 30 | 55.4 | 54 | 33.3 | 461 and 300 |
|  |  | F_MCC | TCCCTGTTCTGACAAGTTTAGTTTGCTT | 28 | 58.5 |  | 39.3 |  |
|  |  | R_MCC | TGAGATCCACATCTATTTCTAGGCATGA | 28 | 57.1 |  | 39.3 |  |
| rs80338943 | *GJB2* | F_GJB | GGACACAAAGCAGTCCACAG | 20 | 59.05 | 53 | 55 | 654 and 358 |
|  |  | FM_GJB | GACACGAAGATCAGCTGCAAGC | 22 | 62.36 |  | 54.55 |  |
|  |  | R_GJB | TGCTTGCTTACCCAGACTCA | 20 | 58.94 |  | 50 |  |
| rs764659822 | *TACR3* | FN_TAC | TGTATGAACCCTGGGGGAAATGTCAGTC | 28 | 66.15 | 56 | 50 | 285 and 181 |
|  |  | RM_TAC | AAAACCAAAGTCATGCCAGGCCGGAT | 26 | 67.48 |  | 50 |  |
|  |  | RN_TAC | TTGATCCCTTGAAACCCAGACTGTCTGC | 28 | 66.71 |  | 50 |  |
| rs1799990 | *PRNP* | FN_PRN | CGCTACCCACCTCAGGGCGGTGGTGGCT | 28 | 76.49 | 60 | 71.43 | 445 and 273 |
|  |  | RM_PRN | GATGGGCCTGCTCATGGCACTTCCCAGAAC | 30 | 72.18 |  | 60 |  |
|  |  | RN_PRN | CCCCCTTGGTGGTTGTGGTGACCGTGTGC | 29 | 74.22 |  | 65.52 |  |
| rs775565634 | *DCC* | FN_DCC | CTCTCTTCATGTGAGGCCCCAGACTAAC | 28 | 66.16 | 56 | 53.57 | 159 and 114 |
|  |  | FM_DCC | GACTCCTCCCTTGAACCCAAACAGCA | 26 | 66.68 |  | 53.85 |  |
|  |  | RN_DCC | ATGGAATAATATCGCTGCTTGCTGTCCA | 28 | 64.94 |  | 42.86 |  |
| rs111033204 | *GJB2* | F_GJB_2 | AGAAGCCGTCGTACATGACA | 20 | 59.11 | 53 | 50 | 509 and 203 |
|  |  | RM_GJB_2 | CACGTGGCCTACCGGAGTCG | 20 | 64.94 |  | 70 |  |
|  |  | R_GJB_2 | TGCTTGCTTACCCAGACTCA | 20 | 58.94 |  | 50 |  |
| rs2814707 | *LOC107987057* | F_LOC | ACTCTCCCATCAACCACAGG | 20 | 59.01 | 53 | 55 | 787 |
|  |  | R_LOC | TTTCTGAGCTGCCTACAGGT | 20 | 58.65 |  | 50 |  |
| rs61745597 | *ZGRF1* | F_ZGR_2 | CCACAGTAGACCTCTGCCTAT | 21 | 58.33 | 53 | 52.38 | 549 |
|  |  | R_ZGR_2 | TCCAAGTTCAACAAATGTAATCCT | 24 | 56.78 |  | 33.33 |  |
| rs201037487 | *FAM98C* | F_FAM | GCAGTGAGGTCTTGAATGGT | 20 | 57.81 | 53 | 50 | 508 |
|  |  | R_FAM | TGGAGAAGTAGCCAGGGACT | 20 | 59.59 |  | 55 |  |
| rs76187047 | *ZGRF1* | F_ZGR | CCAACACCTCAAATAGAAAGC | 21 | 55.12 | 53 | 42.86 | 607 |
|  |  | R_ZRG | CAAGCAATCTACTCGCCTTG | 20 | 56.9 |  | 50 |  |
| rs12478318 | *SCN9A* | F_SCN | GGACCAGTCCTTTCATTTGCC | 21 | 59.45 |  | 52.38 | 533 |
|  |  | R_SCN | GGTGGCACATGAACGACTTC | 20 | 59.48 |  | 55 |  |
| rs751037529 | *PRKN* | F_PRK | ACTGCTCTAGTTCCACGGTAA | 21 | 58.48 | 55 | 47.62 | 569 |
|  |  | R_PRK | AGCACAGTCTACACAACCCT | 20 | 58.58 |  | 50 |  |
| rs200483989 | *SLC3A1* | F_SLC_3 | GCAAAGGATCAGGGAGGGCA | 20 | 62.22 | 59 | 60 | 417 |
|  |  | R_SLC_3 | GAGCTAGGGTGGCAGGTGTT | 20 | 61.84 |  | 60 |  |
| rs114073621 | *PYGM* | F_PYG | CCACCATTAGAGGGCATCCT | 20 | 58.86 | 55 | 55 | 650 |
|  |  | R_PYG | CAGTAGAGCTGACCCCAGAC | 20 | 59.18 |  | 60 |  |

Supplemental Table 2: Sanger sequencing results

| **rsID** | **Gene** | **HGVS** | **Genotype** | **Image** |
| --- | --- | --- | --- | --- |
| rs116040763 | *RIPK1* | *NC_000006.12:g.3113257C>T* | *CT* | **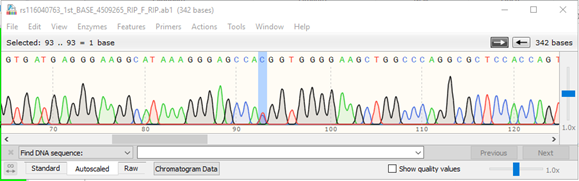** |
| rs200994482 | *SLCO1B1* | *NC_000012.12:g.21224840G>A* | *GA* | 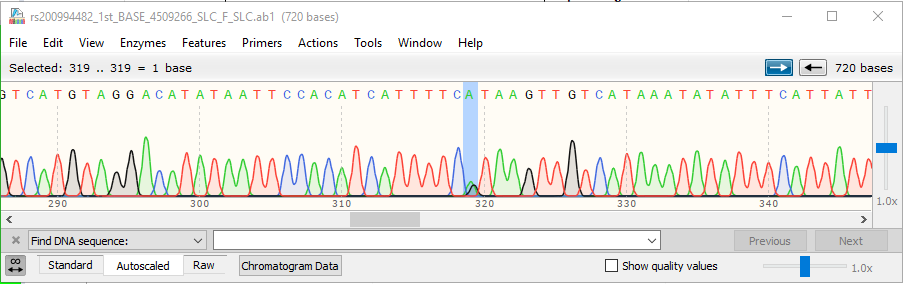 |
| rs779015128 | *ACADSB* | *NC_000010.11:g.123043110delC* | *C-* | 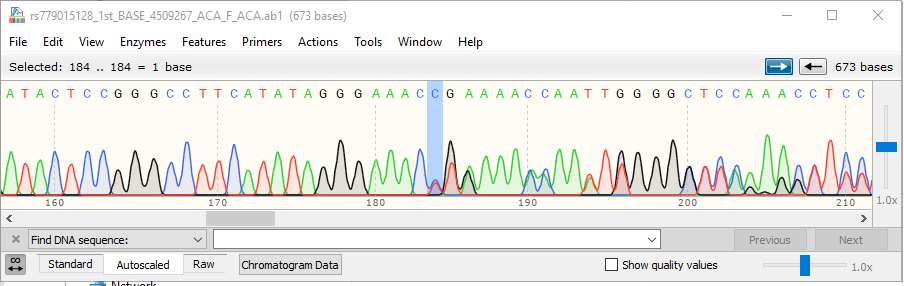 |
| rs587784464 | *TCF4* | *NC_000018.10:g.55350904G>A* | *GA* | 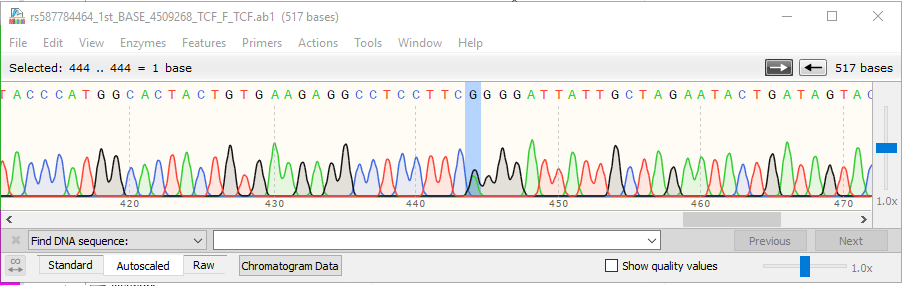 |
| rs2395029 | *HCP5* | *NC_000006.12:g.31464003T>G* | *TG* | 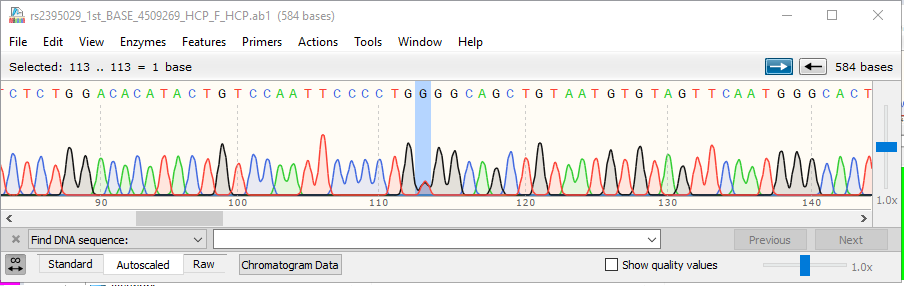 |
| rs139494583 | *KAT6A* | *NC_000008.10:g.41792077C>T* | *CT* | 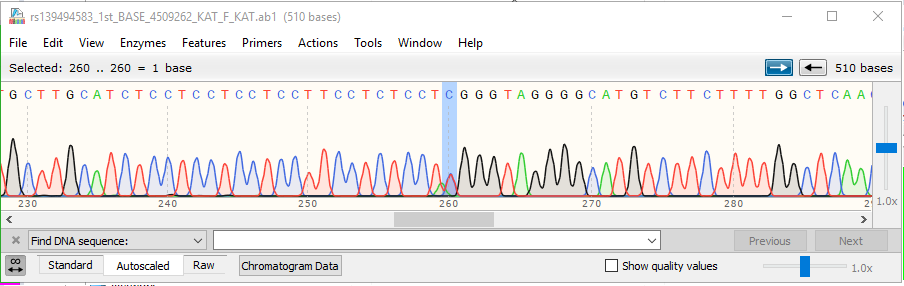 |
| rs750896617 | *MOCOS* | *NC_000018.10:g.36260092C>T* | *CT* | 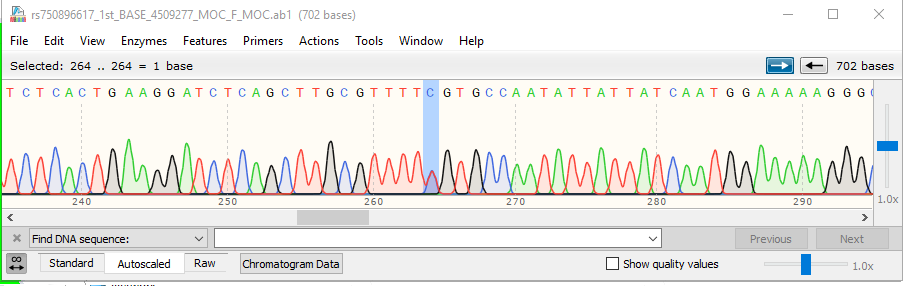 |
| rs9332964 | *SRD5A2* | *NC_000002.12:g.31529325C>T* | *CT* | 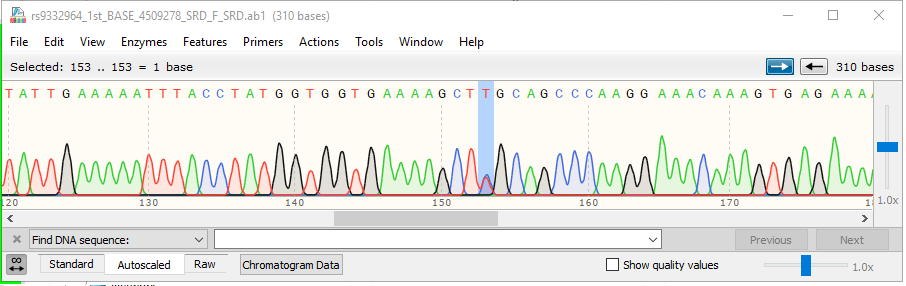 |
| rs143944436 | *CUBN* | *NC_000010.11:g.16940152G>A* | *GA* | 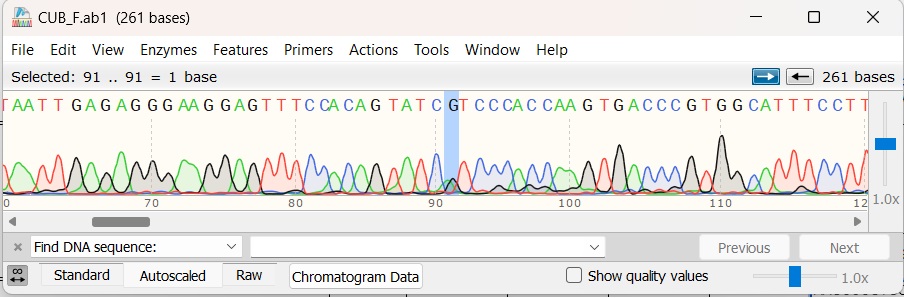 |
| rs119103221 | *MCCC2* | *NC_000005.10:g.71635176C>G* | *CG* | 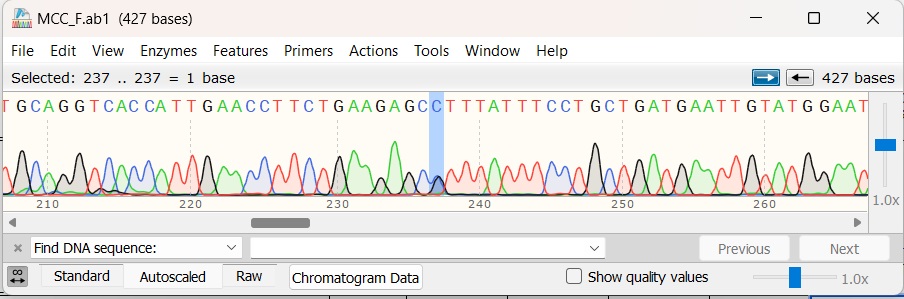 |
| rs80338943 | *GJB2* | *NC_000013.11:g.20189349del* | *G/-* | 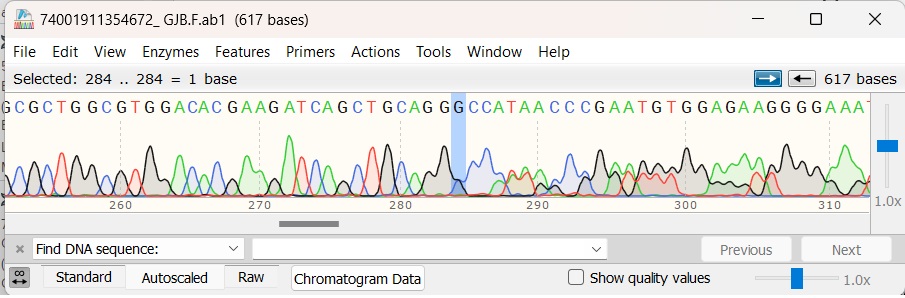 |
| rs764659822 | *TACR3* | *NC_000004.12:g.103658260G>A* | *GA* | 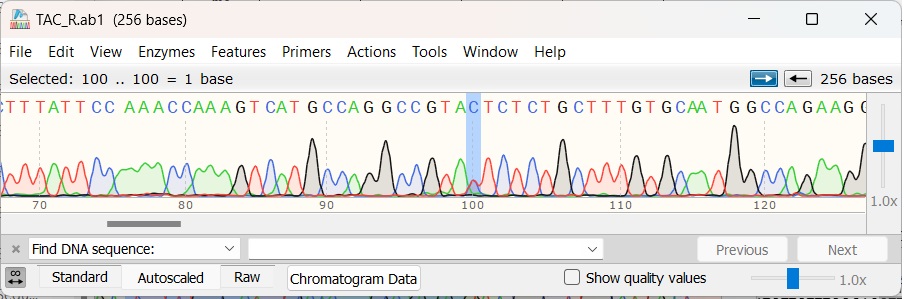 |
| rs1799990 | *PRNP* | *NC_000020.11:g.4699605A>G* | *AG* | 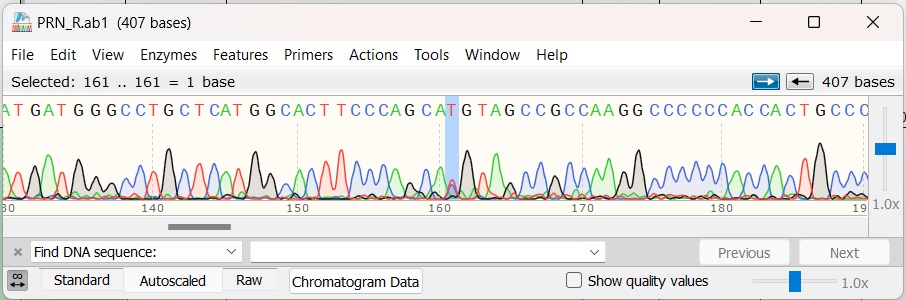 |
| rs775565634 | *DCC* | *NC_000018.10:g.53339808G>A* | *GA* | 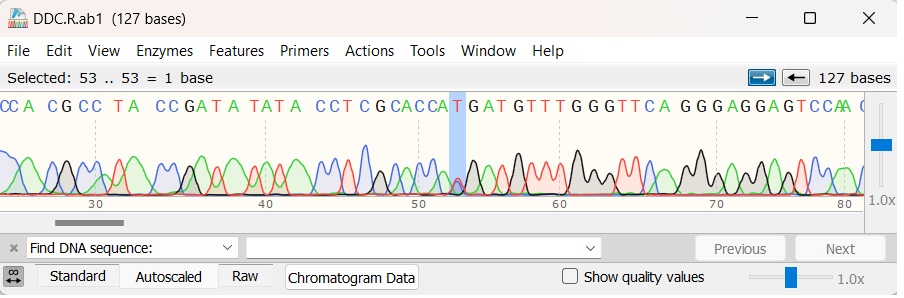 |
| rs111033204 | *GJB2* | *NC_000013.11:g.20189282_20189283del* | *AT/-* | 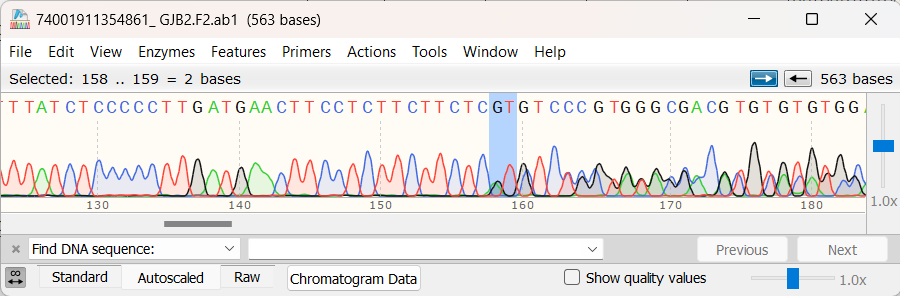 |
| rs2814707 | *LOC107987057* | *NC_000009.12:g.27536399C>T* | *CT* | 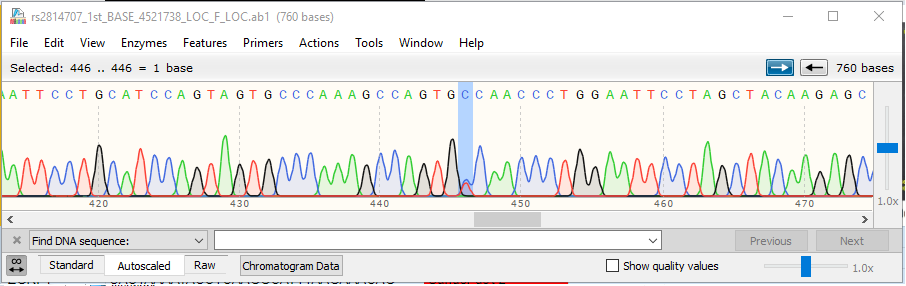 |
| rs61745597 | *ZGRF1* | *NC_000004.12:g.112623837G>T* | *GT* | **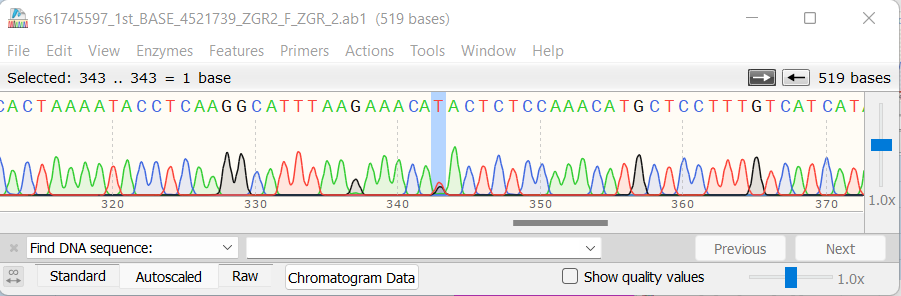** |
| rs201037487 | *FAM98C* | *NC_000019.10:g.38407003C>T* | *CT* | 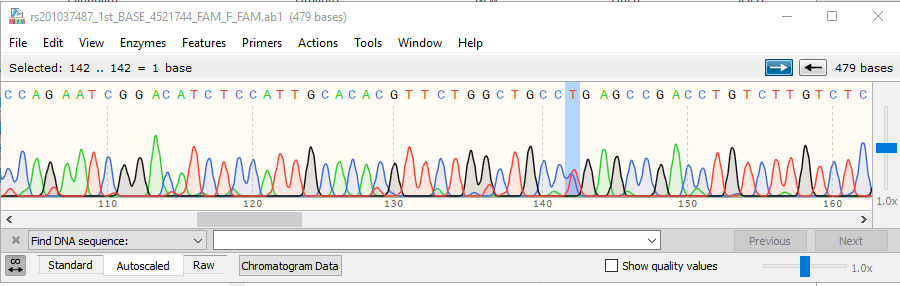 |
| rs76187047 | *ZGRF1* | *NC_000004.11:g.113506711C>T* | *CT* | 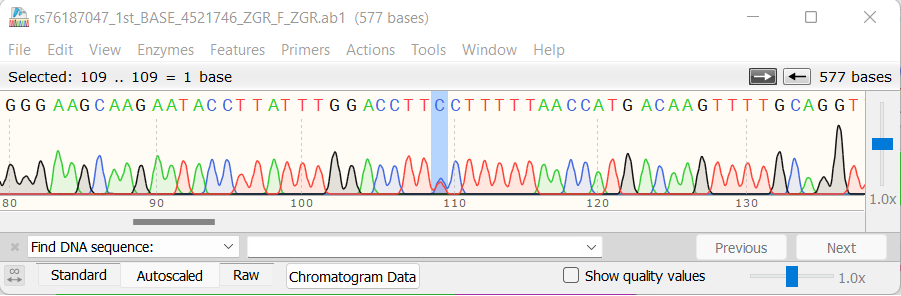 |
| rs12478318 | *SCN9A* | *NC_000002.12:g.166277030T>G* | *TG* | **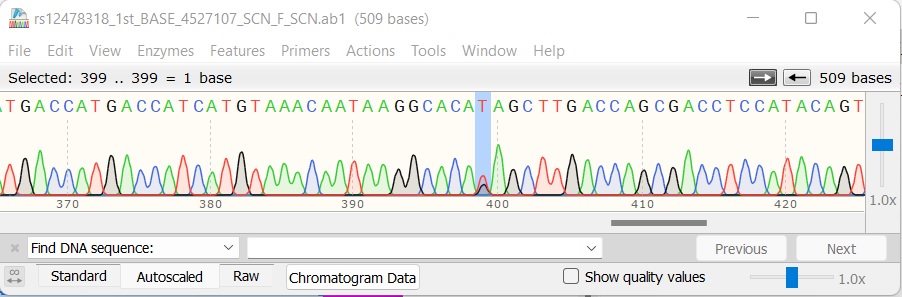** |
| rs751037529 | *PRKN* | *NC_000006.12:g.161785793C>G* | *CG* | **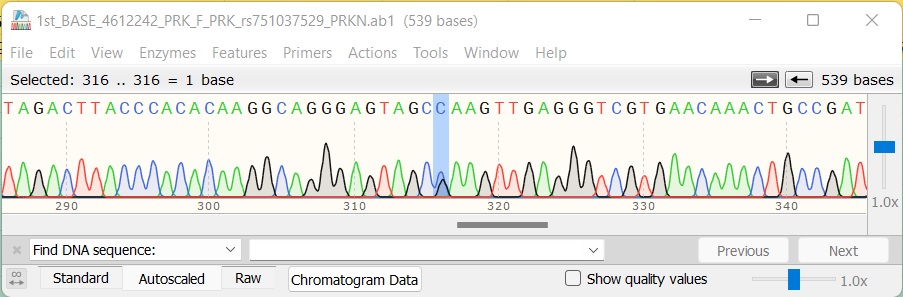** |
| rs200483989 | *SLC3A1* | *NC_000002.12:g.44286074C>T* | *CT* | **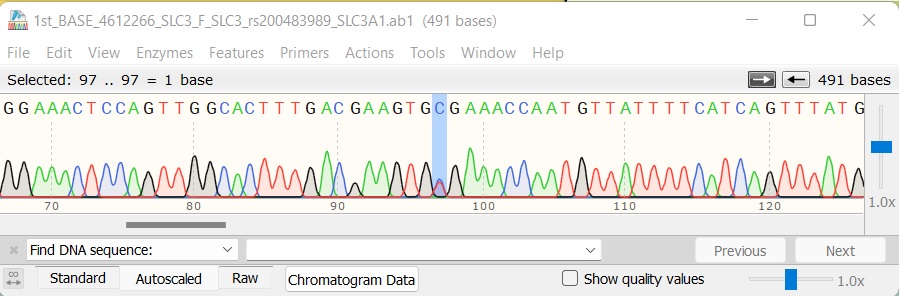** |
| rs114073621 | *PYGM* | *NC_000011.10:g.64751346G>A* | *GA* | **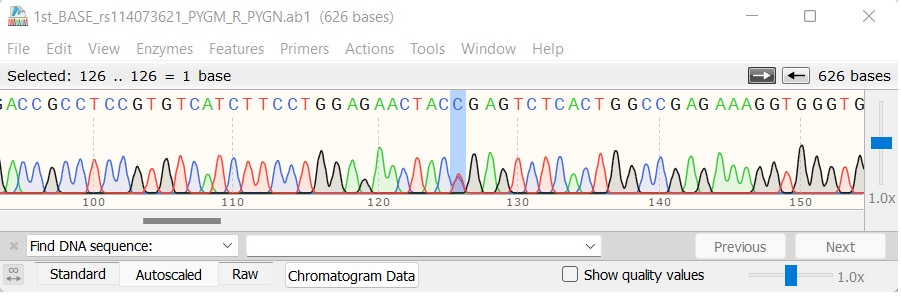** |


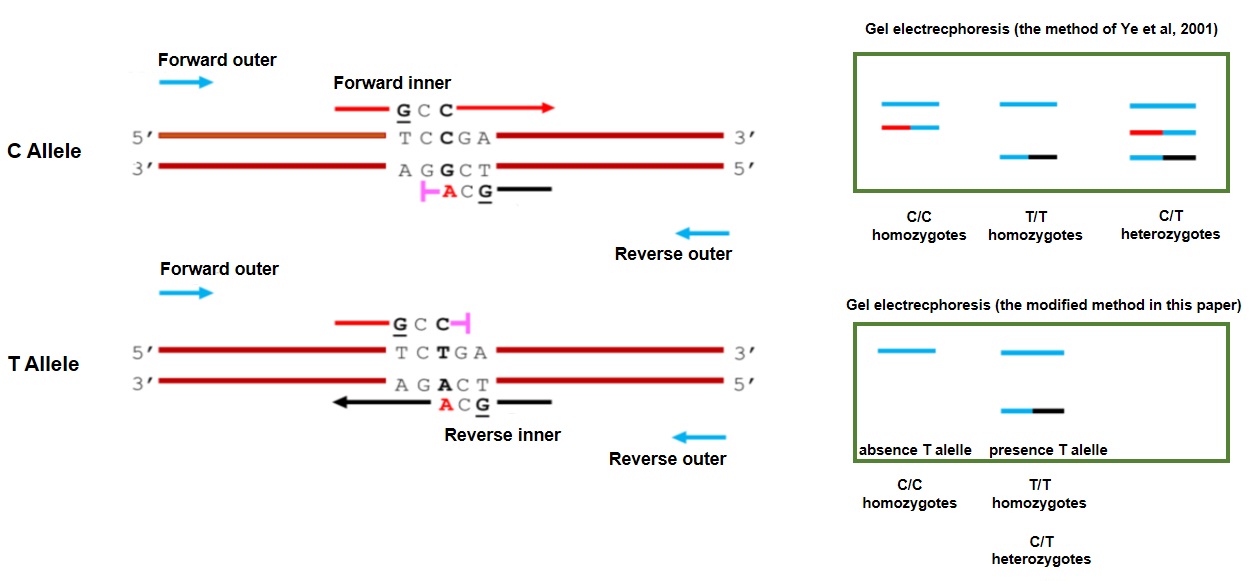


Supplemental Figure 1 Principle of validation of identified variants with ARMS-PCR used in this study. A method from Ye et al (2001) for identifying a point mutation using PCR^[52]^. For example, The C–T substitution, where C is the reference allele and T is the alternative allele, is the single nucleotide polymorphism used here as an illustration. In this study, ARMS-PCR is simply used to validate the presence or absence of an alternative allele (identified pathogenic/likely pathogenic mutation). To amplify the flanking area containing the desired SNP, we designed two outer primers (shown by the blue arrow), but only one inner primer which is specific to the alternative allele (T allele, indicated by the black arrow). This inner primer amplifies the template with the Alternative allele; and does not amplify the template with the reference allele (G allele in this example)
